# Antiobesity Effects of Ginsenoside Rg1 on 3T3-L1 Preadipocytes and High Fat Diet-Induced Obese Mice Mediated by AMPK

**DOI:** 10.3390/nu10070830

**Published:** 2018-06-27

**Authors:** Huimin Liu, Jing Wang, Meihong Liu, Hongyu Zhao, Sanabil Yaqoob, Mingzhu Zheng, Dan Cai, Jingsheng Liu

**Affiliations:** 1 College of Life Science, Jilin Agricultural University, Changchun, Jilin 130118, China; liuhuimin@jlau.edu.cn (H.L.); wangjing11151204@163.com (J.W.); 2 National Engineering Laboratory for Wheat and Corn Deep Processing, Changchun, Jilin 130118, China; liumh@jlau.edu.cn (M.L.); sanabily67@gmail.com (S.Y.); zhengmzhu@163.com (M.Z.); dan1980623@163.com (D.C.); 3 College of Food Science and Engineering, Jilin Agricultural University, Changchun, Jilin 130118, China; 4 Chinese Medicine Science Academy of Jilin Province, Changchun, Jilin 130118, China; fixiov5815@126.com

**Keywords:** ginsenoside Rg1, obesity, adipogenesis, lipogenesis, lipolysis, AMPK

## Abstract

Ginsenosides Rg1 is one of the major pharmacologically active saponins in ginseng, which as an antioxidant reduces oxidative damage in the liver and can also be used to prevent cardiovascular diseases and diabetes. However, there is no research targeting the effect of lipid metabolism in high-fat diet (HFD)-induced mice. In this study, we evaluated the anti-obesity effects of Rg1 in 3T3-L1 adipocyte cells and HFD-induced obese C57BL/6J mice. Administration of Rg1 to HFD-induced obese mice significantly decreased body weight, total cholesterol, and total triglyceride levels. In addition to effects in 3T3-L1 cells, Rg1 reduced the accumulation of lipid droplets in a dose-dependent manner. Furthermore, Rg1 exhibits an anti-adipogenic effect via regulation of the expression of the transcriptional factors and lipid metabolism-related genes in vivo and in vitro. We observed that Rg1 administration significantly increased the phosphorylation level of AMP-activated protein kinase (AMPK) in both epididymal white adipose tissue and 3T3-L1 cells. These results indicated that Rg1 works both in an anti-adipogenic and anti-obesity manner through inducing AMPK activation, inhibiting lipogenesis, and decreasing intracellular lipid content, adipocyte size, and adipose weight.

## 1. Introduction

Recent data from 195 countries has revealed that the prevalence of obesity has doubled in more than 70 countries since 1980; over 600 million adults were obese in 2015 [1]. Obesity has become one of the most serious public health concerns across the globe, as it is associated with increased risk of numerous chronic diseases, including type 2 diabetes (T2D), hypertension, cardiovascular disease (CVD), and cancer [2,3]. Obesity is a multifactorial disease; the main pathway is a sustained state of positive energy balance leading to the excess accumulation and storage of white adipose tissue [4]. Undoubtedly, the ideal treatment strategy involves the appropriate dietary and lifestyle changes. However, it is a great challenge for people to maintain long-term lifestyle modifications. Thus, nutritional interventions to create a negative energy balance, thus reducing the fat stores, represent the most effective way to treat obesity [2].

Adipocytes play a vital role in the progress of obesity in relation to lipid homeostasis and energy balance. 3T3-L1 cells are well-known models for assessing preadipocyte differentiation and lipid metabolism. Preadipocyte differentiation, also called adipogenesis, is regulated by a set of transcription factors including peroxisome proliferator-activated receptor γ (PPARγ), CCAAT/enhancer-binding protein (C/EBP) family members, and sterol regulatory element binding protein 1c (SREBP-1c). Furthermore, these factors can modulate the expression of downstream target genes involved in lipogenesis and lipolysis, such as acetyl-CoA carboxylase (ACC), fatty acid synthase (FAS), fatty acid binding protein (FABP), hormone-sensitive lipase (HSL), and perilipin 1 (PLIN1) [5,6].

Obesity is a disorder related to energy imbalance. AMP-activated protein kinase (AMPK) is a crucial cellular energy sensor. Once activated, AMPK triggers catalytic processes to generate ATP while inhibiting anabolic processes that consume ATP in an attempt to restore cellular energy homeostasis. AMPK is considered as a potential target for the treatment of metabolic disorders. Increasing evidence has demonstrated that AMPK can inhibit adipogenesis and suppress the expression of SREBP-1c, PPARγ, and FAS in adipocytes [7,8].

Ginseng has been widely used to treat diseases for more than 2000 years in Asia. It has been reported that ginseng provides health benefits with respect to CVD, T2D, immune function, and obesity [9,10]. There are various bioactive compounds in ginseng, such as ginsenosides, polysaccharides, peptides, fatty acids, vitamins, and flavonoids. Many reports have indicated that ginsenosides are the major therapeutic constituents, and over 100 ginsenosides have been isolated and identified [9,11]. Recent studies have reported that ginsenosides provide various potential benefits to human health, and can even be used to treat metabolic syndromes [10,12]. Moreover, the antiobesity effect of ginsenosides has been investigated in adipocytes and mice [13,14,15,16]. Ginsenosides Rg1 and Rb1 are the major pharmacologically active saponins, and have been used as the markers for quality control of ginseng products. Rg1 exists both in *Panax ginseng* and *Panax quinquefoliu*, and is especially abundant in the leaves of *Panax quinquefoliu* [9,17,18,19,20]. It has been found that Rg1 can reduce oxidative damage in liver as antioxidants, promote the capability of learning and memory, and can also be used to prevent cardiovascular diseases and diabetes [16,17,21,22]. Additionally, it was shown that Rg1 increased plasma membrane translocation of GLUT4 in C2C12 skeletal muscle cells, and protected mice from dietary-induced obesity via activation of the AMPK pathway [23]. However, there is no evidence of the effects of Rg1 on lipid metabolism in adipocyte cells and white adipose tissue. In this study, we have investigated the effects of Rg1 on the inhibition of adipogenesis in 3T3-L1 cells and in obese mice induced by a high-fat diet (HFD).

## 2. Materials and Methods

### 2.1. Materials

Ginsenoside Rg1 was purchased from ShangHai YuanYe Biotechnology Co., Ltd. (Shanghai, China). Antibodies against PPARγ, AMPKα, p-AMPKα (Thr172), ACC, p-ACC (Ser79) and β-actin were purchased from Cell Signaling Technology (Beverly, MA, USA). HFD was provided by Chinese Medicine Science Academy of Jilin Province, the composition was shown in Table 1.

### 2.2. Animals

Male KM mice (16–18 g) were purchased from Liaoning Changsheng Biotechnology Co., Ltd. (Liaoning, China). Mice were housed in specific-pathogen-free facility cages in standardized conditions: 12 h dark–light cycles, 21 ± 2 °C room temperature, and 45–65% relative humidity. After one week of acclimation, mice were randomly divided into following four groups, each consisting of 12 mice, (1) standard treatment diet (STD) group, fed with a normal chow diet for 8 weeks; (2) HFD group, fed with a HFD for 8 weeks; (3) the L-Rg1 group, fed with HFD for 4 weeks and then fed with HFD and 10 mg/kg Rg1 for another 4 weeks; and (4) the H-Rg1 group, fed with HFD for 4 weeks and then fed with HFD and 20 mg/kg Rg1 for another 4 weeks. Ginsenoside Rg1 was dissolved in 0.5% carboxymethylcellulose sodium and administered at 10 mL/kg daily by gavage. Body weight and length of the mice were recorded and these values used to calculate Lee’s index (body weight (g) 1/3 × 1000/body length (cm)). At the end of the experimental period, the mice underwent fasting for 12 h and were then sacrificed. Blood was obtained from the retro-orbital plexus to collect serum. The adipose tissue and liver were rapidly removed and weighed, rinsed with physiological saline solution, and stored at −80 °C. All experiments and animal were approved by the Institutional Animal Care and Use Committee at the Chinese Medicine Science Academy of Jilin Province (Approval number: SYXK (JI) 2015-0009).

### 2.3. Cell Culture and Differentiation

The 3T3-L1 preadipocyte mouse cells were obtained from the American Type Culture Collection (CL-173, Rockville, MD, USA), and were grown in Dulbecco’s modified Eagle’s medium (DMEM), supplemented with 10% (v/v) newborn calf serum (NCS), and a 1% penicillin–streptomycin mixed solution as an antibiotic in an atmosphere of 5% CO_2_ at 37 °C. For adipocyte differentiation, the 3T3-L1 preadipocyte cells were cultured for 2 days to post-confluence, then were stimulated for 2 days with differentiation medium (DMEM containing 10% fetal bovine serum, 0.5 mM 3-isobutyl-1-methylxanthine, 1 µM dexamethasone, 0.125 mM indomethacin, and 10 µg/mL insulin). Subsequently, the cells were incubated for 4 days with DMEM containing 10% fetal bovine serum and 10 µg/mL insulin. The 3T3-L1 preadipocytes were treated with or without Rg1 during the differentiation.

### 2.4. Oil Red O Staining

Oil Red O was used to stain intracellular lipids as described previously [22]. Briefly, differentiated 3T3-L1 cells in different groups were stained with Oil Red O, and were then fixed with 4% polyformaldehyde for 30 min, followed by staining with fresh Oil Red O solution for 10 min at room temperature. The droplets were dissolved in isopropanol and quantified by measuring the absorbance at 530 nm.

### 2.5. Biochemical Analysis

The levels of serum triacylglycerol (TG), total cholesterol (TC), high-density lipoprotein (HDL), and low-density lipoprotein (LDL) were determined using commercial detection kits (Nanjing Jiancheng of Bioengineering Institute, Nanjing, China). The levels of free fatty acid (FFA) were determined using enzyme-linked immunosorbent assay kit (Bio-Techne China Co. Ltd., Shanghai, China).

### 2.6. Histological Analysis

Epididymal white adipose tissue (WAT) and liver were dissected, washed in saline and immediately fixed in 10% buffered formalin for 24 h and embedded in paraffin. Then, 5-μm sections were prepared and stained with hematoxylin and eosin (H&E). White adipose and liver morphological states were observed; the adipocyte sizes were quantified by a light microscope and images were obtained at ×200 magnification.

### 2.7. mRNA Quantification by RT-PCR

To measure the effect of Rg1 on gene expression in white adipose tissue and 3T3-L1 cells, RT-PCR were performed as previously described [24]. Briefly, total RNA was isolated using RNA iso Plus reagent (TaKaRa, Japan). Complementary DNA was synthesized using the Prime Script RT reagent kit (TaKaRa, Japan). Specific mice primers were designed and shown in Table S1. The mRNA expression levels were normalized using β-actin mRNA and calculated using the 2^−ΔΔ*C*t^ method.

### 2.8. Western Blotting

To measure the effect of Rg1 on gene expression in white adipose tissue and 3T3-L1 cells, western blotting was performed as previously described [24]. Total protein content was determined by the BCA Protein Assay Kit (Vazyme Biotech Co., Ltd., Nanjing, China).

### 2.9. Statistical Analysis

Data were presented as mean ± standard deviation (SD) for all the results. Statistical analyses were evaluated by one-way ANOVA and two-tailed Student’s *t*-test using Prism 6.0 software (GraphPad Software, La Jolla, CA, USA). A *p*-value < 0.05 was considered statistically significant compared with the HFD group or control.

## 3. Results

### 3.1. Effects of Rg1 on Body Weight and Adipose Tissue Mass

**Figure 1:**
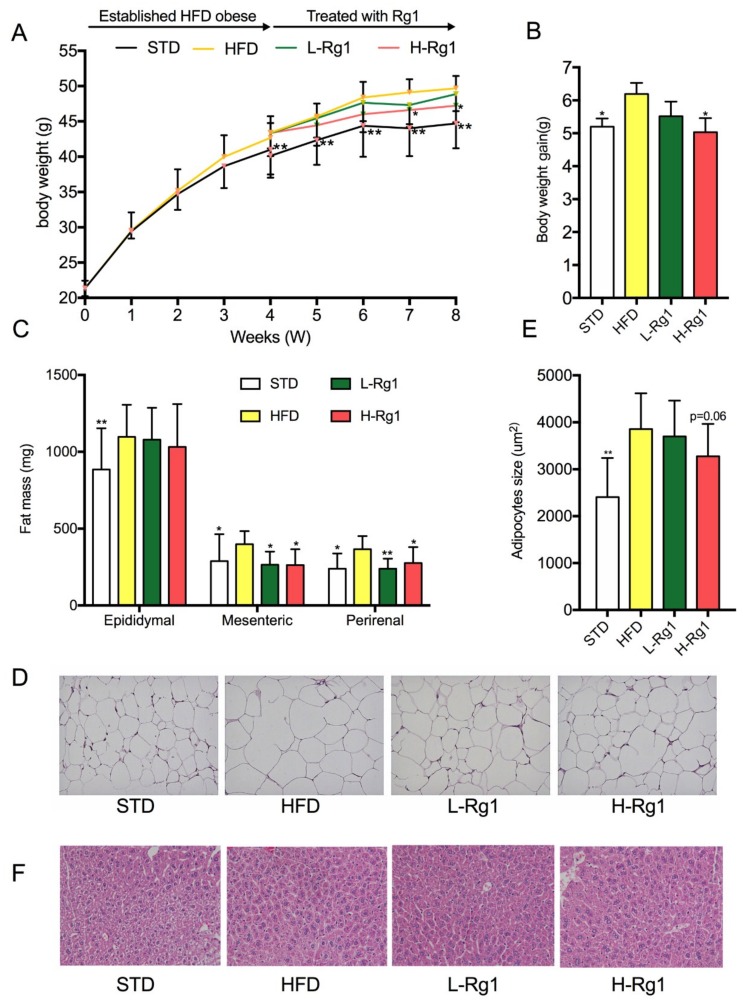
Effect of Rg1 treatment on body weight and adipose mass. (**A**) Body weight; (**B**) Body weight gain in 4–8 weeks; (**C**) Fat mass of epididymal, mesenteric, and perirenal tissues; (**D**) Epididymal adipose tissues stained by H&E; (**E**) Adipocyte size; (**F**) Liver tissue stained by H&E. High and low doses (20 and 10 mg/kg, respectively) of Rg1 were administered from the fourth to the eighth week, and 10 mL/kg 0.5% carboxymethyl cellulose sodium(CMC-Na) solution was used as a vehicle, administered by gavage to STD and HFD groups. Body weight was recorded weekly. STD: standard treatment diet + 0.5% CMC-Na group, HFD: high-fat diet + 0.5% CMC-Na group, L-Rg1: high-fat diet + 10 mg/kg Rg1, H-Rg1: high-fat diet + 20 mg/kg Rg1. Data are represented as means ± standard deviation (SD, *n* = 9). * *p* < 0.05 versus HFD group; ** *p* < 0.01 versus the HFD group. HFD: high-fat diet; H&E: hematoxylin and eosin.

As shown in Figure 1A, after one week of acclimation, the initial body weights in the STD group and HFD group were same. However, after four weeks of high-fat diet, there was a significant difference in body weight between the STD and HFD group, and the high-fat diet obese model was established. Consequently, the HFD group was divided into three groups and treated with Rg1. Then, after four weeks of high-dose Rg1 treatment (20 mg/g), a significant difference was seen in body weight gain as compared to the HFD group (Figure 1B). Moreover, Lee’s index, which is often quoted as a reliable indicator of obesity, was determined after the mouse test, as shown in Table 2. Lee’s index for L-Rg1 and H-Rg1 was clearly lower than for the HFD and STD groups (Table 2).

Furthermore, the effects of Rg1 on adipose tissue and liver were examined. There was no marked difference in the relative adipose weight (Table 2). However, a significant difference was seen in perirenal fat and mesenteric fat compared to the HFD groups (Figure 1C). Although the difference in epididymal fat was not significant, for L-Rg1 and H-Rg1 the levels were slightly lower than in the HFD group. According to the H&E staining of epididymal WAT, adipose size was lower than in the HFD group (Figure 1C,D). For the liver, there was a significant difference in relative weight between the H-Rg1 (32.51 ± 6.43) and HFD groups (37.60 ± 4.40), although there were no obvious pathological changes shown through the H&E staining (Figure 1F). This suggests that Rg1 suppressed the body weight gain by inhibiting the adipose tissue hypertrophy and hyperplasia in HFD obese mice.

### 3.2. Effect of Rg1 on Serum Biochemical Parameters of Mice

As shown in Table 3, mice fed with HFD had higher TG, TC, FFA levels and lower HDL levels compared with the chow diet group, with no significant difference in LDL levels. The results indicated that the HFD mice suffer from significant hyperlipidemia. Moreover, HFD obese mice treated with 20 mg/g Rg1 showed a significant decrease in TG and TC levels, and the FFA levels were lower than in the HFD group (*p* = 0.06). However, there was no effect on HDL and LDL levels.

### 3.3. Effect of Rg1 on Regulated Gene Expression of Lipid Metabolism in Epididymal Adipose Tissue

**Figure 2:**
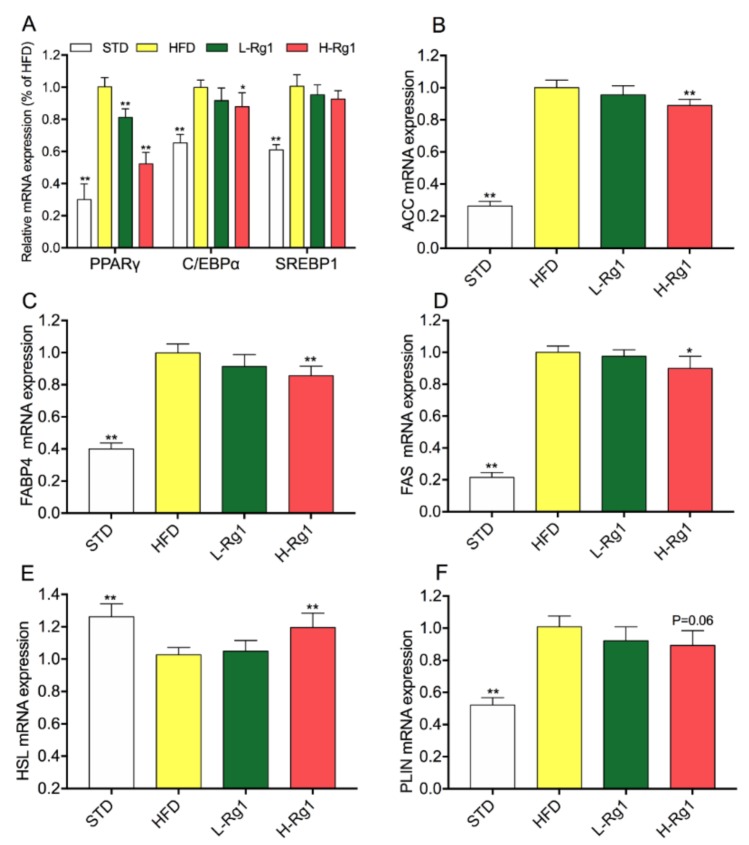
Effect of Rg1 on the expression of adipogenesis-related genes in epididymal white adipose tissue (WAT). Total RNA was extracted from epididymal WAT, and relative mRNA expression levels of adipogenic transcription factors (**A**), and ACC (**B**), FABP4 (**C**), FAS (**D**), HSL (**E**), and PLIN (**F**) were measured using RT-PCR. Data are represented as means ± SD (*n* = 3), * *p* < 0.05 versus HFD group; ** *p* < 0.01 versus HFD group. PPARγ: peroxisome proliferator-activated receptor γ, C/EBP: CCAA/enhancer-binding protein family members, SREBP-1c: sterol regulatory element binding protein 1c, FAS: fatty acid synthase, FABP4: fatty acid binding protein 4, HSL: hormone-sensitive lipase, and PLIN: perilipin 1.

To illuminate the molecular mechanisms underlying the anti-obesity effects of Rg1, transcription levels of specific genes involved in adipogenesis, lipogenesis, and lipolysis were measured by qPCR. As shown in Figure 2A, the expression of adipogenic transcription factors (PPARγ, C/EBPα, and SERBP1) dramatically increased in the HFD group compared with the STD group. Meanwhile, in the L-Rg1 and H-Rg1 groups, the expression of these genes was attenuated, especially for PPARγ. Moreover, the expression of ACC (Figure 2B), FAS (Figure 2C), and FABP4 (Figure 2D) was suppressed with Rg1 administration. This indicated that the lipid synthase was reduced. HSL and PLIN1 are expressed in adipocytes and regulate lipolysis. According to Figure 2E,F, there was a significant difference in the H-Rg1 and STD groups compared with the HFD groups. Together, Rg1 can inhibit adipocyte differentiation and lipid accumulation in epididymal WAT by regulating the expression of related genes.

### 3.4. Effect of Rg1 on Cell Viability and Intracellular Lipid Accumulation in 3T3-L1 Cells

**Figure 3:**
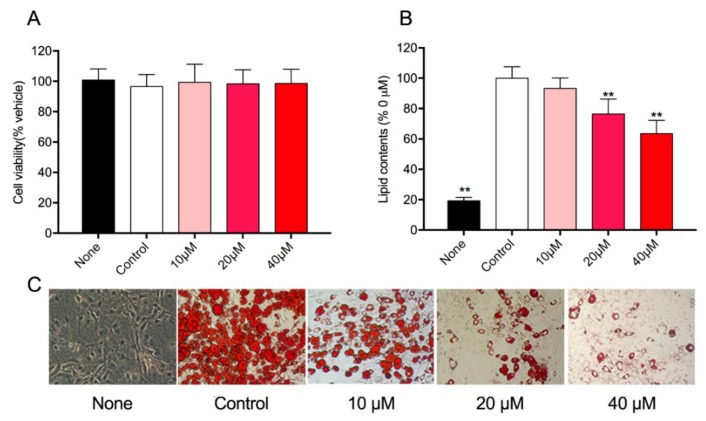
Effect of Rg1 on cell viability and accumulation of lipid droplets in 3T3-L1 cells. (**A**) Cells were treated with different concentration of Rg1 (10, 20, 40 μM) for 72 h and measured by MTT assay. Data are represented as means ± SD (*n* = 3). The 3T3-L1 cells were supplemented with differentiation medium in the presence of Rg1 for 8 days; 0.1% dimethyl sulfoxide was used as a vehicle. The cells were stained with Oil Red O (**C**), and lipid content (**B**) was determined. “None” means cells were cultured in normal medium without Rg1 treatment, and “Control” means cells were cultured in differentiation medium without Rg1 treatment. Data are represented as means ± SD (*n* = 6), ** *p* < 0.01 versus control.

The viability of 3T3-L1 cells treated with different concentration of ginsenoside Rg1 was determined by MTT assay. As shown in Figure 3A, after 72 h of treatment with Rg1, there was no significant cytotoxicity for each concentration. Hence, the concentrations of 10 μM, 20 μM, and 40 μM were used in subsequent research. To investigate the effect of Rg1 on preadipocyte differentiation, the intracellular lipid droplets were stained by Oil Red O and the lipid content was quantified. Interestingly, it was found that Rg1 reduced the accumulation of lipid droplets in a dose-dependent manner (Figure 3C). The total lipid contents of cells treated with 10, 20, and 40 μM of Rg1 decreased to 93.2%, 76.4%, and 63.5%, respectively (Figure 3B).

### 3.5. Effect of Rg1 on the Expression Level of Adipogenic-Specific Genes in 3T3-L1 Cells

**Figure 4:**
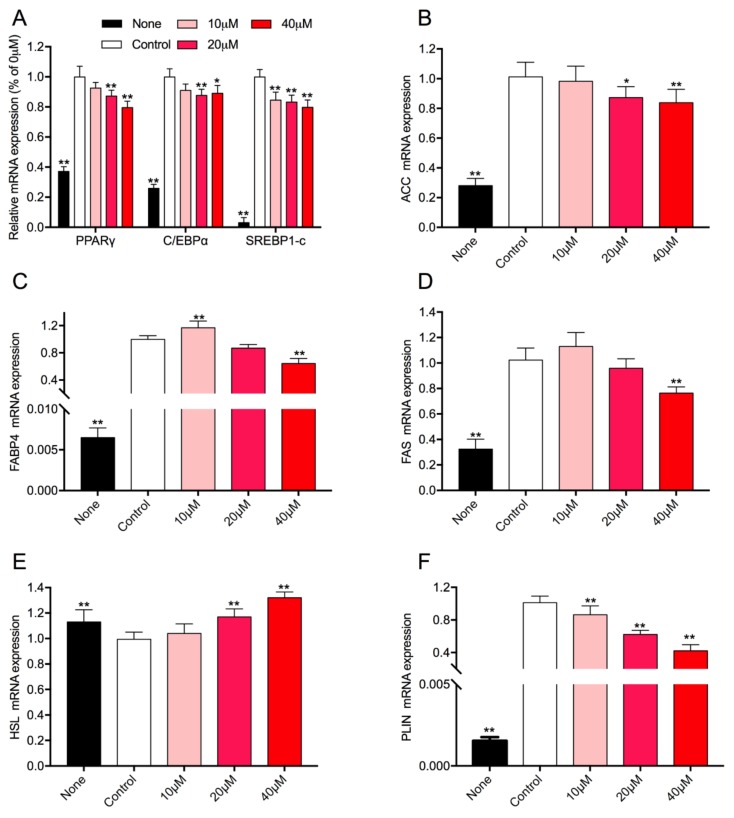
Effect of Rg1 on the expression of adipogenesis-related genes in 3T3-L1 cells. Total RNA was extracted from 3T3-L1 cells, and relative mRNA expression levels of adipogenic transcription factors (**A**), and ACC (**B**), FABP4 (**C**), FAS (**D**), HSL (**E**), and PLIN (**F**) were measured using RT-PCR. Data are represented as means ± SD (*n* = 9), * *p* < 0.05 versus control group; ** *p* < 0.01 versus control group.

3T3-L1 is a cell line used in biological research on the white adipose tissue. To investigate the molecular mechanisms of Rg1 on the preadipocyte differentiation and lipid metabolism, transcription levels of related genes in 3T3-L1 were measured by qPCR. The results indicated that the relative mRNA levels of PPARγ, C/EBPα, and SERBP1 were remarkably suppressed compared with untreated cells (Figure 4A). Moreover, Rg1 also decreased the expression levels of ACC, FABP4, and FAS, which were related to lipid anabolism (Figure 4B–D). Meanwhile, levels of HSL and PLIN (involved in lipid catabolism) were induced and reduced, respectively (Figure 4E,F). Interestingly, the expression levels of FABP4 and FAS were promoted with treatment in the concentration of 10 μM Rg1. In general, Rg1 can inhibit cell differentiation, promote the lipid catabolism, and suppress the lipid anabolism in 3T3-L1 cells, especially with treatment at 40 μM Rg1.

### 3.6. Effects of Rg1 on AMPK Activation in Adipose Tissue and Adipocytes

**Figure 5:**
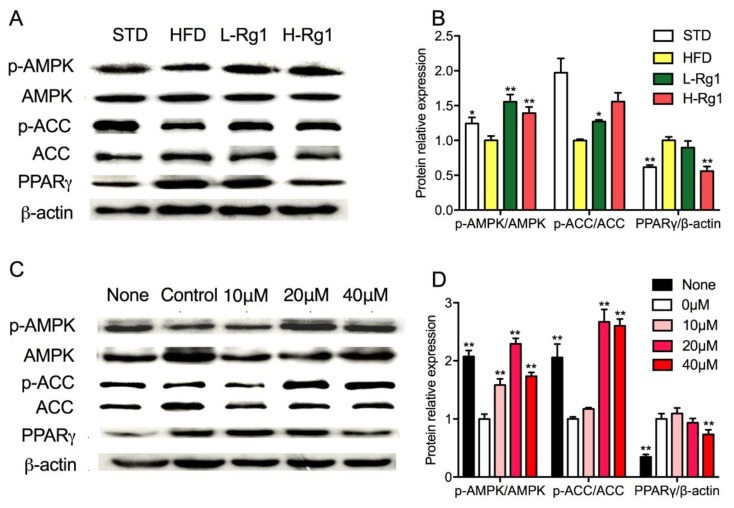
Effect of Rg1 on the activation of AMPK pathway in epididymal WAT and 3T3-L1 cells. The protein expression of AMPK, p-AMPK, ACC, p-ACC, PPARγ (**A**,**C**) and relative protein levels (**B**,**D**) were measured by western blot analysis (The concentrations refer to ginsenoside Rg1). Data was means ± SD (*n* = 6), * *p* < 0.05 versus HFD or control; ** *p* < 0.01 versus HFD or control. “None” means cells were cultured in normal medium without Rg1 treatment, and “Control” means cells were cultured in differentiation medium without Rg1 treatment.

It is well known that AMPK can regulate lipid metabolism as an energy cellular sensor. AMPKα is the predominant catalytic subunit in white adipocytes and can be activated by phosphorylation and enhance catabolism. To investigate whether the activation of AMPK was implicated in Rg1 suppression of adipocyte differentiation and the phosphorylation of AMPKα was determined in vivo and in vitro. It was shown in Figure 5A,B, the protein expression levels of AMPK, ACC, and PPARγ in the HFD group were significantly different compared to chow diet mice. When HFD obese mice were administered Rg1, the protein expression of PPARγ was suppressed, and the phosphorylation of AMPK and ACC were promoted significantly. As in vitro, treatment with Rg1 enhanced AMPK and ACC phosphorylation compared with the control group (Figure 5C,D). For PPARγ, the protein expression was significantly decreased with treatment with 40 μM Rg1. There was no dramatic change in other two groups treated with 10 μM and 20 μM Rg1 (Figure 5D). These results suggested that Rg1 administration could promote AMPK activity to enhance lipid catabolism and suppress ACC activity to decrease the lipid synthase in vivo and in vitro.

## 4. Discussion

The potential anti-obesity activity of ginsenosides has been highlighted in the last few decades. Ginsenoside Rb1 and Rg3 attracted more attention than others. Rb1 treatment significantly reduced body weight gain, body fat content, and fatty liver whereas improved glucose tolerance in HFD-induced obese rats [15,25,26]. It also increased basal glucose uptake and promoted browning in 3T3-L1 adipocytes [27]. It was shown that Rg3 ameliorated HFD-induced obesity by reducing lipid accumulation and total TGs in mice, while it was effective in the inhibition of adipocyte differentiation in cells [28,29,30,31,32]. Moreover, the potential anti-obesity effects of other ginsenosides have also been reported in 3T3-L1 cells and obese mice, such as Rh1 [33], Rh2 [13], F2 [14], compound K [34] and Rb2 [35]. Jinbo Li et al. found that Rg1 could inhibit dietary-induced obesity and improved insulin resistance and glucose intolerance [23]. However, there is no research targeting the effect of Rg1 on lipid metabolism in adipocytes and HFD-induced mice. In this work, we found that four weeks of Rg1 treatment suppressed the body weight gain by inhibiting the adipose tissue hypertrophy and hyperplasia, and reduced lipid accumulation, total TGs, and TCs in HFD obese mice.

Adipocytes play a key role in the progress of obesity. Obesity is characterized by increased adipose tissue mass that results from both hyperplasia and hypertrophy. Hypertrophy is mainly determined by the adipocyte differentiation, which generates mature adipocytes from preadipocytes, and hyperplasia is determined by the balance of lipogenesis and lipolysis [36]. It is well known that PPARγ, C/EBP, and SREBP are the major transcription factors in adipocyte differentiation and lipid regulation [37,38]. We found that Rg1 treatment inhibited the mRNA expression of these transcription factors both in 3T3-L1 cells and adipose tissue of HFD-induced obese mice (Figure 2A and Figure 4A). Additionally, Rg1 could downregulate the protein expression level of PPARγ in a dose-dependent manner both in vivo and in vitro (Figure 5). Therefore, several key enzymes involved in lipid metabolism were examined. ACC, FAS, and FABP4 are the critical enzymes for lipogenesis, while HSL and PLIN are key for lipolysis [38,39]. We found that Rg1 could downregulate the expression of ACC, FAS, FABP4, and PLIN, and upregulate the expression of HSL (Figure 2 and Figure 4). In general, Rg1 inhibited adipocyte differentiation by suppressing PPARγ, C/EBP, and SREBP expression, thus enhancing lipolysis and reducing the lipogenesis.

Recently, the effects of natural compounds to prevent and treat diseases through AMPK activation have attracted researcher attention. As a nutrient and energy sensor, AMPK regulates metabolic energy balance at the whole-body level, so it is considered as a potential target to treat obesity and diabetes [8,40]. AMPK can be activated by the phosphorylation at Thr172, located in a conventional Ser/Thr kinase domain of the α subunit. It is indicated that AMPK activity is reduced in adipose tissue of obese rodents and humans, and nutritional interventions promoted this activity and then prevented the progress of obesity [7,41]. Increasing evidence shows that ginsenosides Rg1 [42], Rg3 [29], compound K [43,44], Rb2 [45], and Re [46] activate AMPK in cells such as HepG2 cells, C2C12 cells, and 3T3-L1 cells, in mice. In accordance with previous work [23], we also found Rg1 could increase the phosphorylation levels of AMPK α1 in vivo and in vitro. AMPK is the main kinase regulator of ACC, which plays an important role in lipogenesis. In our study, it was shown that Rg1 suppressed the activity of ACC through phosphorylation by AMPK. However, the activity of upstream kinases of AMPK is not detected, especially liver kinase B1 (LKB1) and calcium-calmodulin-dependent kinase kinase 2 (CaMKK2). To look deeper into the mechanisms of AMPK, inhibitory or small interfering RNA should be used in the future study to provide sufficient evidence.

In present studies, the doses of Rg1 usually ranged from 10 to 40 mg/kg in animal models, and 10 to 40 μM in cells [16,17,21,23,42,47]. In this work, we investigated the effect of Rg1 at doses of 10 and 20 mg/kg in an animal, and found that both doses could ameliorate HFD-induced obesity, but the effect of 20 mg/kg Rg1 was more significant than 10 mg/kg. In addition, different doses (10, 20 and 40 μM) were employed in the 3T3-L1 cells; we found that Rg1 reduced lipid accumulation and regulated genes expression in a dose-dependent manner, but had a maturation effect on AMPK phosphorylation at doses of 40 μM. A previous paper indicated that a *Panax ginseng* extract (PGE) reduced lipid accumulation at 1 μg/mL [48,49]. Our study shows the same effect of RG1 at 40 μM (32 μg/mL). It is reported that PGE consists of Rg1, Re, Rf, Rb1, Rc, Rb2, and Rd [48]. Hence, a synergistic effect of ginsenosides may provide the more active effect, or perhaps one particular ginsenoside is more effective than Rg1. It suggested that further studies are needed to elucidate the molecular mechanism of specific ginsenoside or combinations. Ultimately, clinical trials will be needed to determine whether the agents such as Rg1 are effective in preventing obesity in human beings.

## 5. Conclusions

In summary, our results suggested that Rg1 significantly reduced obesity in HFD mice. The body weight gain, Lee’s index, and serum TG levels and TC levels were decreased by Rg1 administration. Rg1 exhibited an anti-adipogenic effect by down-regulating the mRNA expression of adipogenic transcription factors (PPARγ, C/EBPα, and SREBP-1c). In addition, Rg1 also activated the AMPK pathway and suppressed ACC activity by phosphorylation, which is related to the suppression of adipogenic differentiation and lipogenesis. These observations are meant to aid in the understanding of the administration of supplementary Rg1 in terms of its potential to prevent or treat obesity.

## Figures and Tables

**Table 1:** Composition of High Fat Diet.

Formula	g/kg
Standard laboratory chow	333.3
Casein	200.0
Hydrogenated coconut oil	220.0
Soybean oil	16.7
Corn starch	102.2
Maltodextrin	102.0
Sodium carbonate	6.7
Potassium citrate	2.7
Choline bitartrate	1.4
Vitamin and mineral mixture	15.0
Total	1000.0

**Table 2:** Effect of Rg1 on body weight and tissue mass in HFD-fed mice.

Groups	STD	HFD	L-Rg1	H-Rg1
Lee’s index	3.49 ± 0.10	3.53 ± 0.07	3.44 ± 0.10 *	3.42 ± 0.15 *
Relative adipose weight (mg/g bodyweight)	34.92 ± 3.90 **	42.60 ± 3.76	41.77 ± 3.00	40.32 ± 4.36
Relative liver weight (mg/g bodyweight)	31.09 ± 5.54 **	37.60 ± 4.40	33.19 ± 6.2	32.51 ± 6.43 *

Data are represented as means ± SD (*n* = 9), * *p* < 0.05 versus HFD group; ** *p* < 0.01 versus HFD group. STD: standard treatment diet + 0.5% CMC-Na group, HFD: high-fat diet + 0.5% CMC-Na group, L-Rg1: high-fat diet + 10 mg/kg Rg1, H-Rg1: high-fat diet + 20 mg/kg Rg1.

**Table 3:** Effect of Rg1 on serum lipid profile in HFD-fed mice.

Groups	STD	HFD	L-Rg1	H-Rg1
TG (mmol/L)	1.06 ± 0.15 *	1.27 ± 0.26	1.12 ± 0.32	0.99 ± 0.23 *
TC (mmol/L)	2.90 ± 0.44 **	3.86 ± 0.33	3.76 ± 0.55	3.10 ± 0.42 *
HDL (mmol/L)	6.20 ± 0.97 **	8.16 ± 1.12	7.47 ± 1.29	8.64 ± 1.15
LDL (mmol/L)	0.42 ± 0.15	0.50 ± 0.22	0.51 ± 0.24	0.68 ± 0.33
FFA (mol/L)	730.28 ± 80.05 *	830.64 ± 125.93	796.29 ± 67.08	785.22 ± 171.90

Data was means ± SD (*n* = 9), * *p* < 0.05 versus HFD group; ** *p* < 0.01 versus HFD group. TG: triacylglycerol, TC: total cholesterol, HDL: high-density lipoprotein LDL: low-density lipoprotein; FFA: free fatty acid.

## Supplementary Materials

The following are available online at http://www.mdpi.com/2072-6643/10/7/830/s1, Table S1: Primers sequences used in qRT-PCR analysis.
