# Supplementary material for: Antiobesity Effects of Ginsenoside Rg1 on 3T3-L1 Preadipocytes and High Fat Diet-Induced Obese Mice Mediated by AMPK
Source: Nutrients. 2018 Jun 27;10(7):830. doi: 10.3390/nu10070830 (PMC6073290; doi:10.3390/nu10070830)
Supplement: Supplementary file 1 [file nutrients-10-00830-s001.pdf]

nutrients

Article

Antiobesity Effects of Ginsenoside Rg1 on 3T3-L1 Preadipocytes and High Fat Diet-Induced Obese Mice Mediated by AMPK

Huimin Liu [1,2], Jing Wang [1], Meihong Liu [2,3], Hongyu Zhao [4], Sanabil Yaqoob [2,3], Mingzhu Zheng [2,3], Dan Cai [2,3] and Jingsheng Liu [2,3,*]

1 College of Life Science, Jilin Agricultural University, Changchun, Jilin 130118, China; liuhuimin@jlau.edu.cn (H.L.); wangjing11151204@163.com (J.W.)
2 National Engineering Laboratory for Wheat and Corn Deep Processing, Changchun, Jilin 130118, China; liumh@jlau.edu.cn (M.L.); sanabily67@gmail.com (S.Y.); zhengmzhu@163.com (M.Z.); dan1980623@163.com (D.C.)
3 College of Food Science and Engineering, Jilin Agricultural University, Changchun, Jilin 130118, China
4 Chinese Medicine Science Academy of Jilin Province, Changchun, Jilin 130118, China; fixiov5815@126.com
* Correspondence: liujs1007@vip.sina.com; Tel.: +86-431-8453-2886

Received: 16 May 2018; Accepted: 21 June 2018; Published: 27 June 2018

Supplementary File

Table S1. Primers sequences used in qRT-PCR analysis.

Gene	Forward primers	Reverse Primers
PPARγ	AGACCACTCGCATTCCTTT	CCACAGACTCGGCACTCAAT
C/EBPα	ACCGGGTTTCGGGACTTGA	CCCGCAGGAACATCTTTAAGTGA
HSL	TCCTGGAACTAAGTGGACGCAAG	CAGACACACTCCTGCGCATAGAC
Perilipin	GTCAATGAACAAGGGCCCAAC	CACAGGCAGCTGCAGAACTCTC
FABP4	TGGGAACCTGGAAGCTTGTCTC	GAATTCCACGCCCAGTTTGA
FAS	TGCTGTTGGAAGTCAGCTATGAA	GATGCCTCTGAACCACTCACAC
SREBP1-c	CCGAGATGTGCGAACTGGA	GAAGTCACTGTCTTGGTTGTTGATG
ACC	AGTGATGGTGGCCTGCTCTTG	AGCAGACGGTGAGCGCATTA
β-actin	CATCCGTAAAGACCTCTATGCCAAC	ATGGAGCCACCGATCCACA
